# Nasal cavity vascular leiomyoma - case report and literature review

**DOI:** 10.1016/S1808-8694(15)30766-7

**Published:** 2015-10-19

**Authors:** Victor Eulalio Sousa Campelo, M.C. Neves, M. Nakanishi, R.L. Voegels

**Affiliations:** 1 2nd year ENT Resident - HCFMUSP; 2 ENT Resident - Department of Otorhinolaryngology - University of São Paulo Medical School - F.M.U.S.P.; 3 MD. Graduate Student Department of Otorhinolaryngology - University of São Paulo Medical School - F.M.U.S.P.; 4 Associate Professor - Department of Otorhinolaryngology - University of São Paulo Medical School - F.M.U.S.P Studied carried out at the Department of Otorhinolaryngology - University of São Paulo Medical School - F.M.U.S.P

**Keywords:** leiomyoma, nasal neoplasms, vascular leiomyoma, nose

## Abstract

Liomyomas of the nasal cavity and paranasal sinuses are rare. They make up less than 1% of all leiomyomas in the human body. This is due to the paucity of smooth muscle in the nose. They are classified in three groups: leiomyoma, angiomyoma and epithelioid leiomyoma. Only 15 cases of vascular leiomyomas have been found in the literature. The treatment of choice is surgical excision. Hereby we present a new case and review the literature.

## INTRODUCTION

Leiomyomas are benign smooth muscle tumors, more commonly found in the uterus (95%), skin (3%), gastrointestinal and food intake tracts (1.5%).^1^^,^^3^ Less than 1% happen in some head and neck structure.^1^ Maesaka et al. (1966) described the first case of nasal vascular leiomyoma.^1^^,^^3^^,^^7^^,^^8^^,^^11^ Since then, 15 cases of nasosinusal vascular leiomyomas have been described, five of them in the inferior turbinate.^1^^,^^11^ We hereby present a case of vascular leiomyoma, its treatment and literature review.

## CASE REPORT

**Figure 1:**
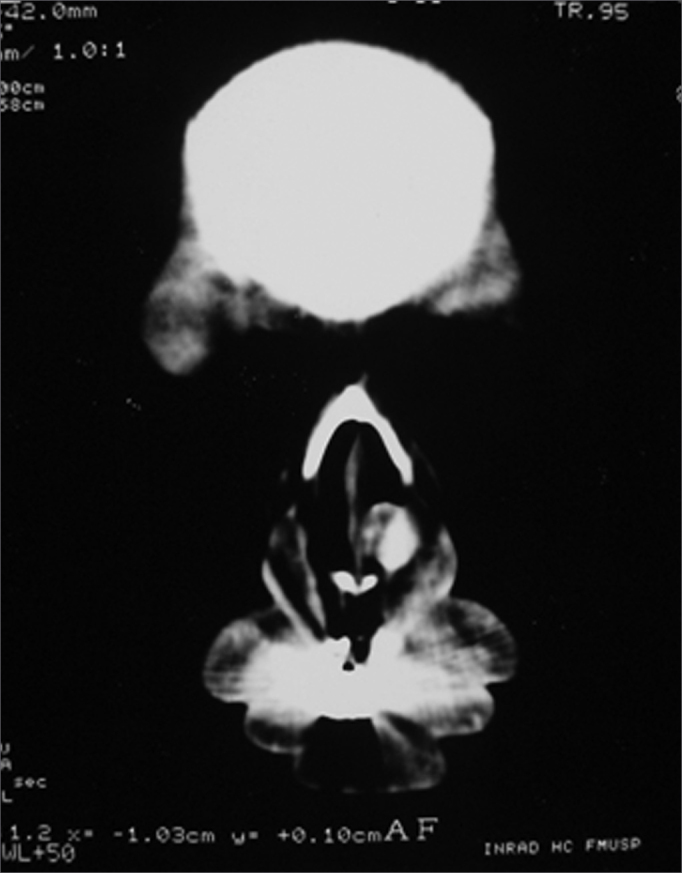
Round nodular lesion, well outlined in the left piriform opening, with soft tissue lining, presenting intense highlight after contrast injection, with 1.2cm in diameter.

V.L.S, 44 years, female, brown, came to the Otolaryngology Department of the University of São Paulo complaining of a red tumoral mass she had for about 4 years. The lesion grew slowly with small volume epistaxis episodes. After 3 years she developed nasal obstruction in he left side and local pruritus. In the physical exam and under nasal endoscopy we noticed a red lesion inserted on the left turbinate head, well outlined, measuring about 1 × 2cm, partially obstructing the nasal cavity. CT scan showed a round nodular lesion, which reacted intensely to contrast injection, having 1.2cm in diameter (Figures 1A and 1B). We biopsied the lesion and the pathology report classified it as a leiomyoma. She was then submitted to endoscopic resection of the lesion and the turbinate's head under general anesthesia. We removed an irregular dark red and elastic fragment measuring 2.2 × 0.9 × 0.7cm. Under light microscopy we observed a nodular lesion made up of relatively organized bundles of smooth muscles, permeated by thick wall vessels formed by smooth muscle cells concentrically aligned with partially patent lumens. We also observed areas of myxoid transformation.

She did not have any complications in her postoperative and has been disease-free for 10 months now.

**Figure 2:**
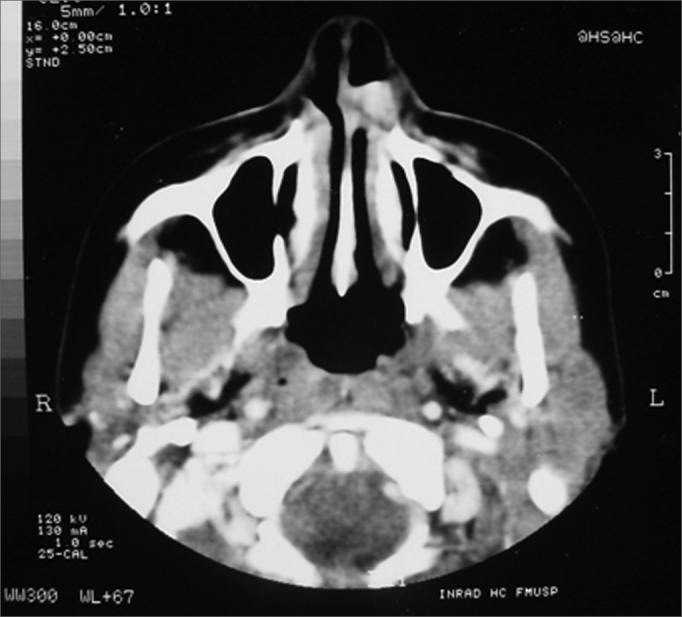
Round nodular lesion, well outlined in the left piriform opening, with soft tissue lining, presenting intense highlight after contrast injection, with 1.2cm in diameter.

## DISCUSSION

Nasal cavity and paranasal sinuses vascular leiomyomas are extremely rare, making up less than 1% of all human leiomyomas. This rarity is partially due to the scarcity of smooth muscle in the nasal cavity, except for the vessels' walls.^1^^,^^14^ In the nasal cavity, we can find smooth muscle tissue in the blood vessels' walls or in the hair erecting muscles of the anterior vestible.^10^ The most accepted theories for these neoplasias advocate that these lesions stem from the proliferation of blood vessels' walls' cells, from the hair erecting muscles or some aberrant undifferentiated mesenchima.^6^

Histological classification of tumors by the World Health Organization^3^^,^^10^ divided the leiomyomas in three groups: leiomyoma, angioleiomyoma (vascular leiomyoma or angiomyoma) and epithelioid leiomyoma (bizarre leiomyoma and leiomyoblastoma). Angiomyomas may stem from surface or deep tissue. In both cases, the neoplasia seem to develop from the vessels' smooth muscle.^11^ Vascular leiomyomas are made up of bundles of relatively organized smooth muscle cells, permeated by thick walled vessels. The surface lesions are made mainly by thick wall vessels associated with proliferative muscle tissue. Deeper lesions are usually larger, probably because of a delay in detection, and frequently present varied histological alterations which are not seen on the surface types. These alterations include an increase in cell number and build up of myxoid substance. We can also observe fibrosis, calcifications and giant cells reaction.5,11 Morimoto (1973) classified these tumors histologically into three types: (i) capilary or solid, (ii) cavernous and (iii) venous. In the limbs, these tumors are mainly solid; while in the head and neck they are frequently of the venous type.8,11 Histopathological differential diagnosis includes hemangioma, angiofibroma, fibromyoma, leiomyoblastoma, angiomyolipoma, vascular leiomyosarcoma.^11^

There are reports of malignant tumor variants of this line. Lack of mitosis seems to indicate the tumor's benign characteristics.^11^ Many dyes and immunohistochemical tests have been used to identify vascular leiomyomas, including desmine, vimentin, Masson's tricomono, actin and myosin. However, this spectrum of methods is not necessary for the diagnosis. In our patient, hematoxylin-eosin dye showed findings very characteristics for angioleiomyoma.

These tumors grow slowly and may persist for a long time.^1^ According to the literature, the most common symptoms are (considering the present case): nasal obstruction (56.25%), epistaxis (56.25%), facial pain (25%) and headache (25%).^1^, ^2^, ^3^, ^4^^,^^6^, ^7^, ^8^, ^9^, ^10^, ^11^, ^12^, ^13^

Comparing literature data, we observed that the most frequent location of the vascular leiomyoma in the nasal cavity has been in the inferior turbinate. Of the 15 cases described (Table 1), 5 originated in the inferior turbinate; and as to symptoms, the initial complaint was nasal bleeding. In a similar fashion, the present case also originated in the inferior turbinate and presented epistaxis as initial symptom.

**Table 1:** vascular leiomyoma cases in the nasal cavity

Authors	Year	Gender	Age	Symptoms	Site	Treatment
Maesaka et al.	1966	F	49	Facial pain	Vestibule	Excision
Wolfowitz et al.	1973	F	42	Epistaxis	Inferior turbinate	Excision
Schwantman et al.	1973	M	57	Nasal obstruction, Headache	Sphenoid sinus, ethmoid and maxillary	Excision
Timiryaleev12	1973	F	25	Epistaxis, Nasal obstruction, Headache	Septum	Excision
McCaffrey et al.13	1978	F	76	Epistaxis, Nasal obstruction	Inferior turbinate	Excision
Daisley14	1987	F	32	Nasal obstruction, Headache	Middle turbinate	Excision
Hanna et al.3	1988	F	64	Epistaxis, Nasal obstruction, Facial pain	Inferior turbinate	Excision
Sawada15	1990	F	41	Nasal mass	Vestibule	Excision
Ragbeer and Stone8	1990	M	49	Epistaxis, Facial pain	Nasal fossa floor	Excision
Khan et al.4	1993	F	71	Nasal obstruction	Inferior turbinate	Excision
Ardekian et al.1	1993	F	54	Epistaxis, Nasal obstruction, Facial pain	Nasal Septum	Excision
Nall et al.5	1996	F	43	Epistaxis, Nasal obstruction, Headache	Superior turbinate	Embolization and excision
Murono et al.17	1998	F	69	Epistaxis	Inferior turbinate	Endoscopic excision
Bloom et al.2	2001	F	50	Nasal obstruction, Headache	Nasal Septum	Endoscopic Excision
Bloom et al.2	2001	F	70	Asymptomatic	Nasal septum	Endoscopic Excision
Present Case	2003	F	44	Epistaxis, Nasal obstruction	Inferior turbinate	Endoscopic Excision

According to Barr et al.^2^ the high incidence of leiomyomas in the inferior turbinate may be attributed to the large local quantity of contractile vascular tissue in the smooth muscle.

Of the 15 cases aforementioned, 13 were women; making up a female predominance of 87.5% if we include the present case.^1^^,^^8^^,^^11^

At the time of diagnosis, the patients' ages ranged from 25 to 76 years, with an average of 55.6 years.^1^^,^^3^^,^^7^^,^^8^^,^^11^

Radiologic studies, such as CT scan or MRI do not help much in diagnosis, however it is important to establish the extension of the lesion and treatment planning.^6^

Treatment for these tumors is based on local resection, and there are no reports of recurrence after total excision.^3^^,^^7^^,^^8^^,^^11^ Bloom (2001) reported a case of spontaneous tumor recurrence after thorough surgical excision. This event shows potential recurrence after incomplete resection and the need for complete excision in order to guarantee a definitive treatment. Surgical approach choice, by nasosinusal endoscopic surgery or by lateral rhinotomy, will depend on tumor location and extension, as well as the need for a better bleeding control.^3^^,^^6^^,^^8^ Our patient underwent endoscopic resection of the lesion under general anesthesia. The procedure was well tolerated and without complications. She is now in her tenth month of postoperative without complications or signs of recurrence.

## FINAL REMARKS

Nasal cavity vascular leiomyomas are extremely rare. The exact origin of this neoplasia is still uncertain, however it is believed that it comes from the muscle cells present in blood vessels' walls. Resection is the procedure of choice and it has high cure rates. The endoscopic procedure is a good option for small to moderate extension tumors.
